# Investigating attentional scope as a novel indicator of emotional state in animals

**DOI:** 10.1038/s41598-022-21151-1

**Published:** 2022-10-19

**Authors:** Anne Hamlaoui, Linda Keeling, Oliver Burman, Else Verbeek

**Affiliations:** 1 Department of Animal Environment and Health, Swedish University of Agricultural Sciences, Box 7068, 750 07 Uppsala, Sweden; 2 School of Life Sciences, University of Lincoln, Lincoln, UK

**Keywords:** Psychology, Behavioural ecology, Physiology, Emotion, Reward, Attention

## Abstract

In humans, contrasting emotional states can lead to a broadening or narrowing of attentional scope. Whether this is also the case in animals has yet to be investigated. If confirmed, measurement of attentional scope has potential as a novel cognitive method of welfare assessment. In this study, we therefore aimed to investigate a test of attentional scope as a measure of emotional state in animals. We did this by inducing four putatively different emotional states in dogs (N = 10), varying in valence (positive, negative) and arousal (high, low), in two different reward contexts (food rewards in Experiment 1, social rewards in Experiment 2) and then assessing dogs’ behavioural responses in a test of attentional scope. We also recorded heart rate variability (HRV) parameters as additional confirmatory affective indicators. In Experiment 1, the dogs showed a narrowing of attentional scope after the induction of both positively valenced emotional states. That dogs were in a positive state was supported by the reduced Standard Deviation of normal-to-normal R-R intervals (SDNN) and the reduced Low Frequency (LF) and Very Low Frequency (VLF) HRV. In Experiment 2, when responses to social rewards were examined, we did not detect any statistically significant differences in attentional scope between the emotional states, but dogs had a slightly narrow attentional scope in the negatively valenced emotional states. The LF tended to be reduced in the high arousal positive treatment. In conclusion, our study provides the first indication that emotional states can also alter attentional scope in animals. The results justify further investigation of this approach for use in animal welfare assessment, although additional studies are needed to refine predictions.

## Introduction

In humans, and more recently also in animals, it has been shown that there is a strong bi-directional link between cognition and emotion^1^. Emotions are defined as brief sensations, elicited by a specific event, and are associated with distinct physiological, behavioural and cognitive reactions^2,3^. Particular emotional states can lead to either a narrowing or a broadening of attention in humans^4^. A narrowing of attention can be described as ‘not seeing the forest for the trees’, while a broadening of attention describes ‘seeing the whole forest’. However, it has not yet been investigated if a similar broadening and narrowing of attentional scope also occurs in animals when they experience different emotional states and, if so, whether it could provide a novel cognitive indicator of animal welfare.

Attentional scope can be measured with a global–local preference test, which uses visual stimuli consisting of one large element, called a global element, which is constructed from several smaller elements, called local elements. A broad attentional scope is defined as a preference for the global elements, while a narrow attentional scope is defined as a preference for the local elements. Anxious people are quicker to detect the local elements in a global–local preference test (i.e. they have a narrow attentional focus) compared to non-anxious people^5,6^. Whereas people in a positive emotional state generally show a broadening of attentional scope^7,8^. A narrow attentional scope ensures that irrelevant stimuli and perceptions can be ignored as individuals attempt to achieve a goal (e.g., get access to a resource or escape from danger)^4,8,9^. However, a constantly narrowed attention towards one type of stimulus can make it harder to shift attention to other stimuli that may be needed more, or when new unforeseen stimuli become available^10^. On the other hand, the broad attentional scope that arises when experiencing positive emotions is associated with more open and holistic processing, which may facilitate detection of new information and the exploration of new opportunities^11^. The ability to narrow and widen attentional scope depending on current emotional state is therefore essential in order to respond to different situations appropriately.

Beyond the valence of the emotion, attentional scope in humans has been shown to be influenced by motivational intensity^4^: positive emotions arise when people are motivated to approach rewarding stimuli (e.g., excitement) but also when they are successful in avoiding negative stimuli (e.g., relief). Negative emotions occur when people experience and/or anticipate harmful stimuli (e.g., fear) and when positive stimuli are removed (e.g., frustration)^12^. In the animal welfare literature, emotions are often described by a two dimensional model consisting of a valence (positive to negative) and arousal (high to low) axis^13^. The concept of motivational intensity and arousal are closely linked: emotions that are highly motivating are often also highly arousing (e.g., anticipation of a food reward) although there are suggestions in the human literature that this is not always the case^4,14^. Nevertheless, for simplicity, and since in this paper we are presenting a study with animals, we have chosen to use the term ‘arousal’ instead of the term motivational intensity.

In summary, attentional scope reflects an interaction between the valence of the emotion and the level of arousal associated with it, so nuancing the response in a particular situation. In humans, it has been shown that while the experience of having achieved monetary gains (low arousal positive emotion) is associated with a broad attentional scope, anticipating monetary gains (high arousal positive emotion) led to a narrowing of attentional scope^15^. Furthermore, even if individuals’ attentional scope narrowed when they had been shown a picture of an appetizing dessert, it became even more narrow when arousal increased because they were led to believe that they would eat the dessert^16^. Regarding negative emotions, high arousal emotions such as disgust^17^ and anxiety^18^ led to a more narrow attentional scope than low arousal emotions such as sadness^17^.

A local/global preference test has already been adapted for animals in order to compare cognitive processing across species (e.g.^19,20^). However, these have been carried out without manipulation of the emotional state. Most non-human primates tend to have a narrow attentional scope^21–23^. Whereas other animal species such as honeybees^20^, fish^24^ and birds^25,26^ have a local preference (but see^27^ for an exception). Dogs preferences for local or global information varied across individuals, which suggests a large individual variation in dogs^19^.

In order to obtain additional measures of emotional state that can aid in interpreting the behavioural data and validate the induction of the different emotional states, we also recorded heart rate (HR) and heart rate variability (HRV) parameters^28^. An increase in HR is generally interpreted as an increase in arousal in both humans^29^ and in dogs^30^, although HR may also indicate valence in humans^31,32^. HRV indexes are often interpreted as indicative of the valence component of emotional states. For example, decreases in Standard Deviation of Normal-to-Normal R-R intervals (SDNN), Root Mean Square of Successive Differences (RMSSD), High Frequency (HF) and Low Frequency (LF) HRV have all been shown to indicate positive valence in dogs^30,33^, while an increase in RMSSD indicated negative valence^33^, although see^34^.

**Figure 1:**
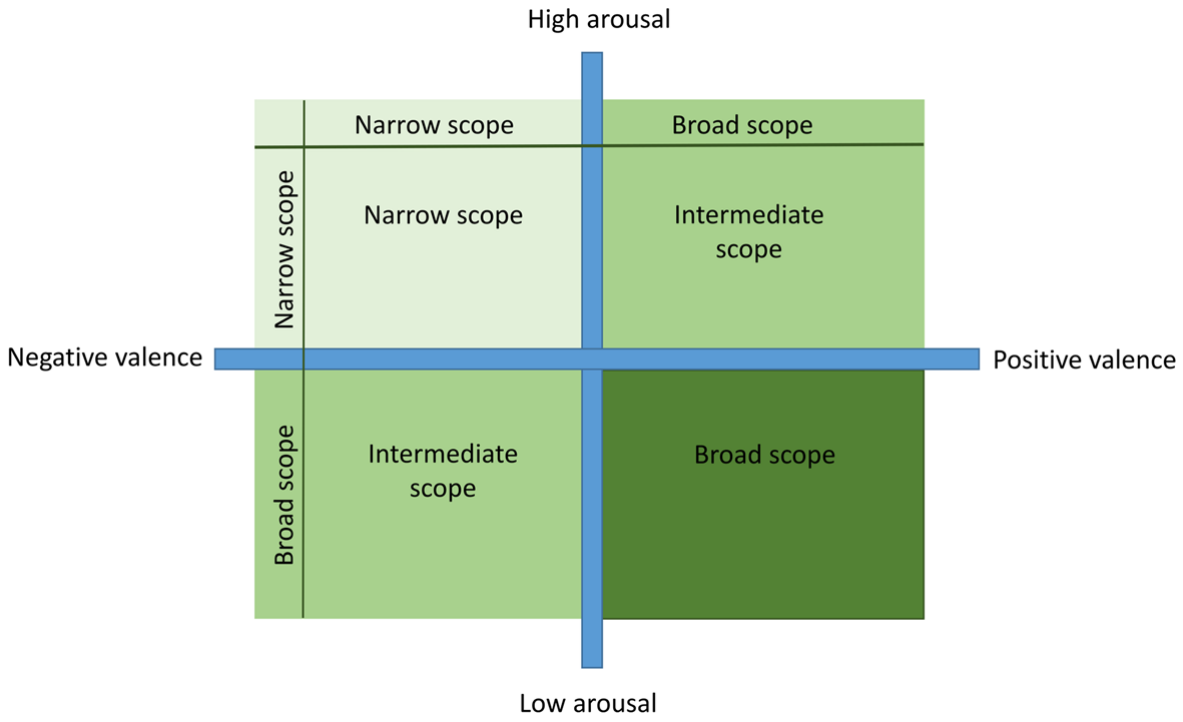
Predictions for the narrowing or broadening of attentional scope within the two-dimensional animal emotional state framework consisting of a valence and arousal axis.

The aim of this study was to investigate the attentional scope methodology as a novel indicator of emotional state in animals. We used dogs, as they have previously been shown to be a suitable model to investigate cognitive and emotional processes (e.g., see^19,35^). We aimed to induce different emotional states varying in valence (positive, negative) and arousal (high, low) in two contexts that are known to be rewarding for dogs; food rewards and social interaction^30,36–38^. We hypothesized that positive emotions in general would lead to a broader attentional scope compared to negative emotions. Furthermore, we hypothesized that arousal would also influence attentional scope, with high arousal states leading to a narrower attentional scope compared to low arousal states. What is less clear is how these interact. Thus, we only make two specific predictions. These are that dogs in a positive, low arousal state will be most broad in their attentional scope and dogs in a negative, high arousal state will be most narrow. We hypothesize that dogs in a positive high arousal state, or a negative low arousal state should be intermediate, but this will depend on the relative contribution of valence and arousal (Fig. 1). In this study we also measure HRV indexes, predicting that heart rate should be higher in the high arousal states and that the valence of the emotion is associated with different HRV indexes, thus validating our inductions of the emotional states.

## Materials and methods

All methods were carried out in accordance with relevant guidelines and regulations. The study was approved by the Uppsala Animal Ethics Committee (document number 5.8.18-01758/2020) and complied with the ARRIVE guidelines^39^.

### 1. Subjects

We estimated that a sample size of around 16 dogs would have been sufficient to address our research question, according to our initial power calculations (with a 95% confidence interval and 80% power) based on an attentional scope pilot study. Therefore, the study started with 21 laboratory beagle dogs to allow for some dogs to drop out due to not reaching the learning criteria.

Dogs that did not succeed in learning the task were excluded (see criteria in the section below). Consequently, five males and five female dogs between 2 to 7 years of age completed the whole experiment. All dogs were kept in kennels at the University Animal Hospital in Uppsala, Sweden, in groups of 3 to 6 individuals. They were kept in indoor enclosures of 24.3 m^2^ (9 m × 2.7 m) from 16:00 h until 8:00 h and in outdoor enclosures varying between 145 and 200 m^2^ (around 6 m × 25 m) the rest of the time. They were fed twice a day with Hill´s Science plan Mature Adult Chicken between 4 and 8 dl per day depending on the dogs’ weight and water was available ad libitum. Twice a week they were walked by a caretaker.

### 2. Global–local preference test stimuli

**Figure 2:**
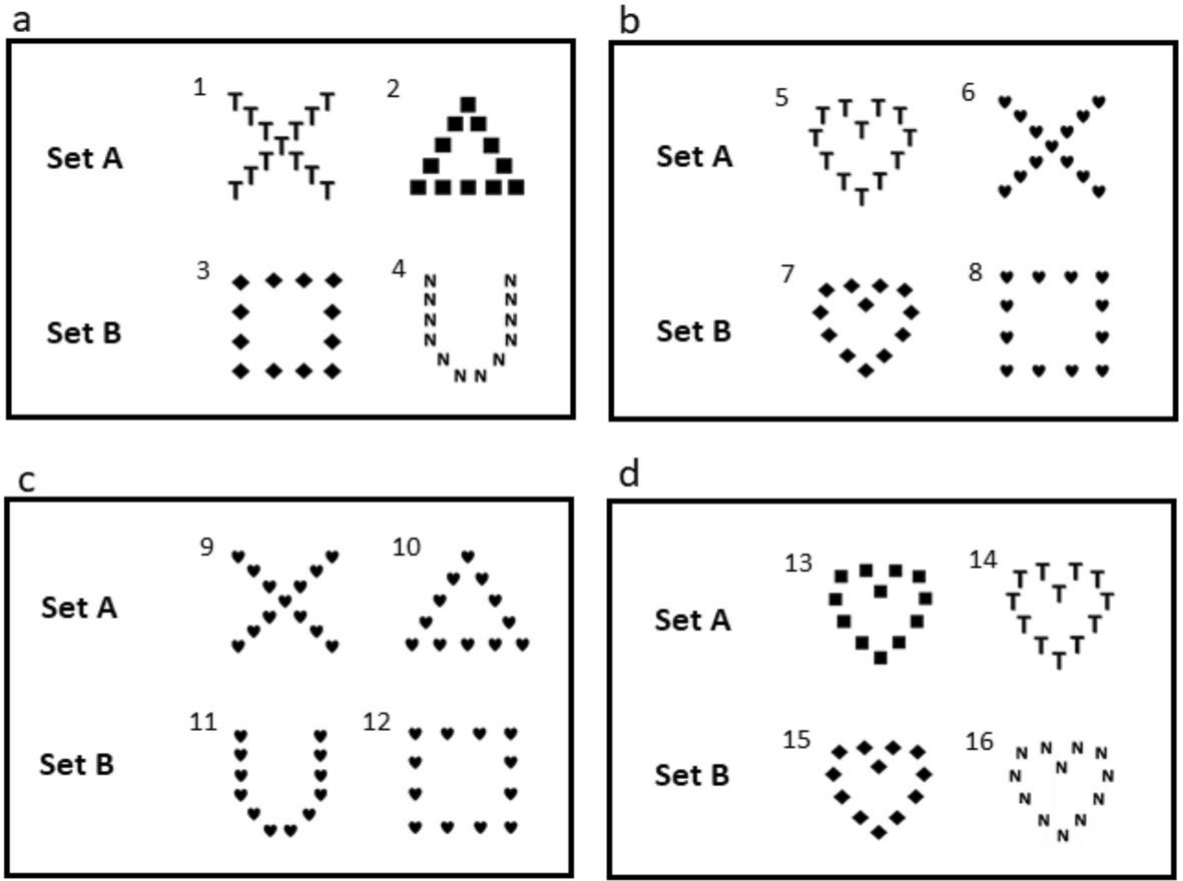
Sets of symbols, with (**a)** showing the base symbols the dogs were trained to discriminate: symbols 1 and 3 were the rewarded symbols, and 2 and 4 were the non-rewarded symbols. (**b)** The conflict task symbols, symbol 5 and 7 indicate local choices and 6 and 8 indicate global choices. (**c)** Symbols used to determine discrimination at the global level only (symbol 9 and 12 are correct), and (**d)** shows symbols for assessing discrimination at the local level only (symbol 14 and 15 are correct).

The glocal-lobal preference test used to assess attentional scope involved training dogs to discriminate between different visual stimuli characterized by a two-level hierarchal pattern. A stimulus was made of a global shape composed of smaller local shapes, which led to two possible levels of discrimination: local or global. The stimuli we used were similar to Navon-type stimuli^40^. The local shapes were 4 cm high and wide, the global shapes were 22.9 cm high and wide. The stimuli were created in PowerPoint (Windows 10), using black opaque symbols on a white background. Two different sets of stimuli were made in order to avoid any bias coming from a specific aspect of the pattern of the stimuli^41^. The training stimuli are shown in Fig. 2a, and half of the dogs were randomly assigned to set A and the other half to set B. We used the random number generator function in Microsoft Excel (function = Rand()) to assign the dogs to the different symbol sets. The conflict stimuli are shown in Fig. 2b, and these were used to assess local and global preferences.

### 3. Experimental setting

**Figure 3:**
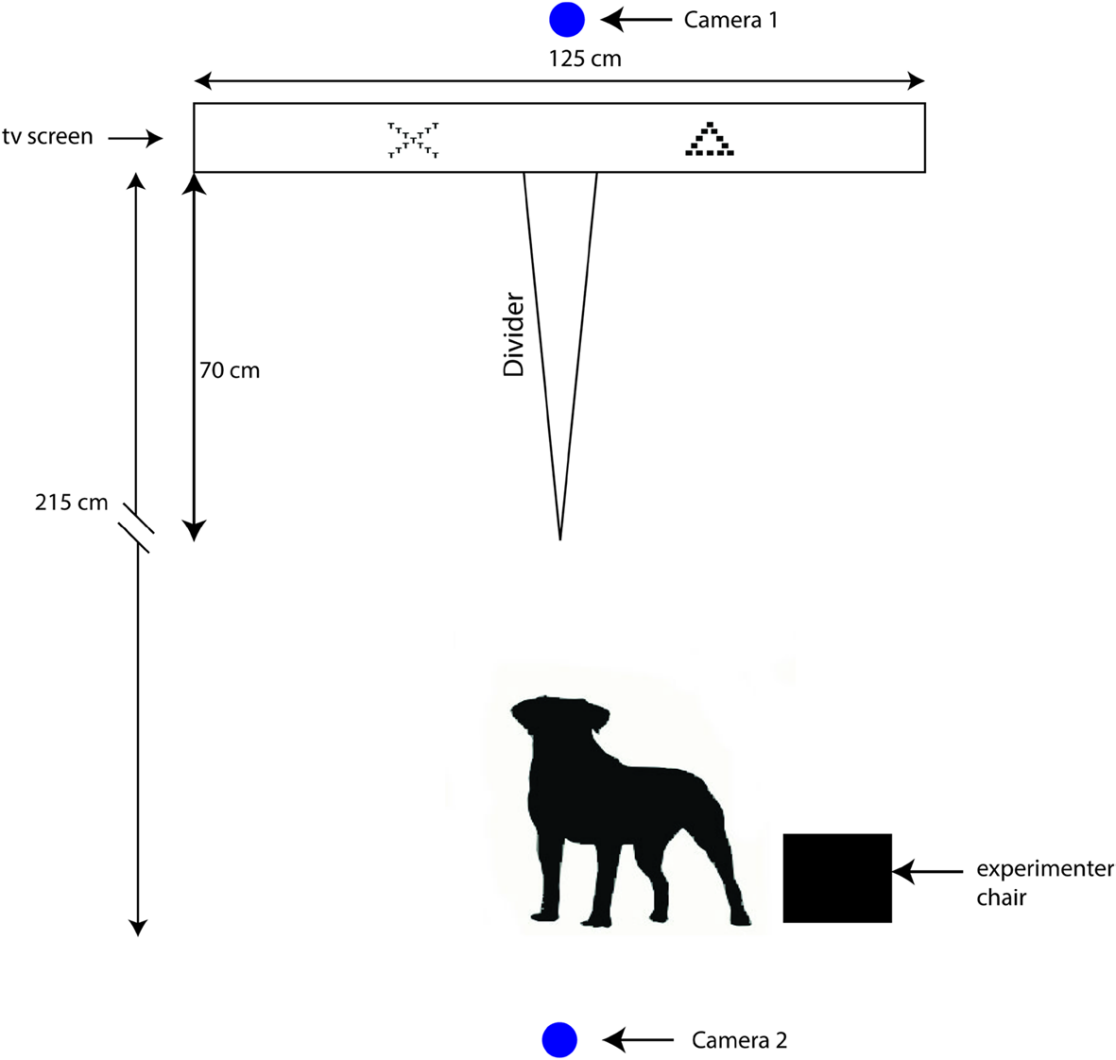
Experimental set-up to assess attentional scope. Dog vector from clipartbest.com.

The experiment was conducted in a separate room close to the dogs’ home pens (1 min walk), and was initially unfamiliar to the dogs. Dogs were gradually habituated to the room, before any training and testing took place. The test apparatus consisted of a TV screen NEC Edition (125 cm long and 72 cm high) and the bottom edge of the screen was 10 cm above the floor level. The speaker (Extron) was placed directly above the screen (aligned to the middle of the screen). A divider of 70 cm long was placed in front of the TV screen to separate it in two halves to help the dog go to one side or another and touch only one symbol with its nose. A chair was placed 2.15 m away from the screen for the researcher to sit on while training and testing the dogs (Fig. 3). The visual angle (visual angle = 2*arctan(0,5*object size/distance)) from the starting position was 5.58 and the visual angle from the beginning of the divider (70 cm in front of the screen) was 28.6., with the object size being 22.9 cm).

### 4. Training

#### Habituation

Dogs were first habituated to the researchers in their home pens, and later to the test room. They were then taught to associate a click on a standard clicker device with a food reward (a mixture of cooked ham and dry dog biscuits and in a ratio of 1 to 3). Following this, the dogs were trained to touch with their nose a single rewarded stimulus (see Fig. 1a) printed on an A4 paper on the floor first and later on the wall, using clicker training. Dogs were not food deprived before training or testing.

#### Training for the global–local preference test

Dogs were trained to either set A or B in sessions of 20 trials for a maximum of three times per day for five days a week. Within each session, the rewarded symbol was displayed 10 times on the left side and 10 times on the right side of the screen in pseudo-random order (no more than three times in a row on the same side). Dogs had to give 16 out of 20 correct responses on two consecutive days to proceed to the next training phase.

The training for the global–local preference test was divided into four different phases in order to gradually teach the dogs to discriminate between the different symbols. In phase 1, the rewarded symbol was displayed on one side of the TV screen while the other side was left blank. In phase 2, the rewarded symbol was displayed on one side of screen and the other side simultaneously displayed one local element of the unrewarded symbol. In phase 3, the rewarded symbol was displayed versus the unrewarded symbol, which was reduced to 50 percent of its total size. In training phase 4, both rewarded and unrewarded symbols were displayed at full size (Fig. 1a). There was no maximum number of sessions in each training phase, but we stopped training dogs that had not reached the learning criteria when the ten dogs that had successfully reached the criteria had completed the full experiment.

At the beginning of each trial, the dog was held by its collar by the experimenter sitting on the chair. Each trial started with the display of a plus sign (1.7 cm wide and 2.1 cm high) in the middle of the screen combined with a ‘pling’ sound to draw the dogs’ attention to the screen. If the dog did not look at the screen within approximately 10 s, the sound was repeated once. Once the dog was looking at the screen, the experimenter closed her eyes, and then the rewarded and unrewarded symbols were displayed simultaneously (Fig. 1a). After about 2 s the experimenter said ‘go’ and released the dog. The experimenter kept her eyes closed until the dog was released in order to avoid influencing the dog’s subsequent choice. The experimenter opened her eyes again once the dog had started to move towards the screen. The dog received a clicker confirmation sound followed by a food reward given by the experimenter if it approached the screen and touched the rewarded symbol. If the dog approached and touched the unrewarded symbol, no click and reward were given, and a new trial was started immediately. This same procedure was then repeated for the other trials.

### 5. Global–local preference test following different treatments

All testing took place between 9:00 h and 11:00 h. Before starting the test, each dog needed to correctly respond to 4 out of 5 final training trials (80% correct). If the dog did not pass this criterion, they received another 5 training trials to pass the criterion. If the training criterion was passed, the dog experienced a treatment designed to induce a specific emotional state. Each experiment was divided into two repetitions: dogs were first tested in all four emotional treatments in a random order (using excel random number generator) in repetition 1, and this was then repeated again using the same random order in repetition 2. All dogs completed repetition 1 before being tested in repetition 2. The reason for the repetition was to balance the side of the rewarded symbol on the screen, to avoid the results being confounded by a side bias or preference for one side of the screen/room, in case these were to exist. Therefore, in each emotional treatment, the rewarded symbol was shown once on the left and once on the right side of the screen in random order in the conflict, local and global discrimination tests (see below).

The dogs performed no more than one test per day. It was not possible for the experimenters to be blind to the treatments, because these had to be administered immediately before testing.

#### Emotional treatments

The four emotions we aimed to induce varied according to valence (positive, negative) and arousal (high, low) in two different reward contexts, according to the four different quadrants of the animal emotion framework^13^ leading to specific predictions regarding attentional scope (see Fig. 1). The high arousal positive states were created by giving the dog a small amount of something it desired^30^, but without letting the dog consume it until satiated, in order to induce (more) desire or excitement (positive and high arousal). In experiment 1, food rewards were used to induce the emotions. We used a hidden treat toy (“Dog Smart”, Nina Ottosson, Sweden) that consisted of 9 holes containing treats that were covered with lids. The dogs had to remove the lids with their mouth or paws in order to access the treats (Table 1). In experiment 2, the emotions were induced by social interactions with the experimenter (Table 2).

**Table 1:** Treatments used in experiment 1 that were designed to induce the different emotions.

Treatment	Emotion (arousal/valence)	Description of the treatment	Duration
Initial access to treats	High arousal positive	Access to the hidden treat toy containing 9 pieces of 2–3 mm sausage	50–60 s
Prolonged access to treats	Low arousal positive	Access to the hidden treat toy containing 9 pieces of dry dog biscuits. The toy was refilled every two minutes, ten times in a row	20 min
Inaccessible treats	High arousal negative	Non-accessible toy with 2–3 mm pieces of sausage, without lids, covered by a wire rack so the treats were visible but not accessible	60 s
No treats	Low arousal negative	Access to an empty hidden treat toy without lids. Wait until the dog stops interacting/loses interest in toy, then wait 30 s	40–60 s

**Table 2:** Treatments used in experiment 2 that were designed to induce different emotions.

Treatment	Emotion (arousal/valence)	Description of the treatment	Duration
Initial access to interaction	High arousal positive	The experimenter played and interacted with the dog	60 s
Prolonged access to interaction	Low arousal positive	Experimenter petted the dog (voluntary) until the dog relaxed (head down on floor/paws without movement, eyes open or closed) or lost interest and walked away	15–40 min
Inaccessible play	High arousal negative	The test dog is alone in the testing area. The experimenter played with a pen mate on the other side of the fence in front of the test dog, and invited the test dog to come to play by calling it and making eye contact with it. However, the dog was unable to cross the fence and join the play session	60 s
No play	Low arousal negative	The experimenter initiated play for 10 s, then stopped, crossed her arms, stood still and ignored the dog. The experimenter waited until the dog stopped interacting, and then tested after 30 s	40–60 s

The low arousal positive states were induced by providing the dogs with rewards until satiated (social context) or until nearly satiated in the food context, in order to create a post-consummatory state of satisfaction or contentment (positive and low arousal)^42^. It was not possible to give food treats until the dogs stopped consuming them, because then they were no longer motivated to perform the test itself (which was identified as a potential issue in a pilot study prior to the experiment).

All positive treatments were dog-led, and dogs were never forced to eat the treats, to play or to be petted. We carefully observed each individual dog to determine when to end the session in the low arousal treatment in experiment 2. The moment that the dog stood up and walked away (which we assumed was because the dog had lost interest) or when the dog completely relaxed (some dogs even fell asleep) were taken as points to end the treatment and test the dog. This time point varied between 15 and 40 min, as indicated in Table 2.

The high arousal negative states were created by preventing the dogs to access a desirable reward, in order to induce immediate frustration^35^. The low arousal negative states were induced by making the dog expect a reward, but never presenting this reward (Tables 1 and 2). In the low arousal negative states, we observed the behaviour of each individual dog and waited until the dogs stopped searching for the reward. We then tested the dogs following a 30 s delay from the point of losing interest. We predicted that these treatments would initially induce frustration but would be followed by disappointment once they had given up searching for the expected reward^43^, with disappointment being lower in arousal than frustration^44^.

All dogs first completed experiment 1 before continuing to experiment 2. It took about 8 days per experiment (1 test per day), so a total of 16 days to complete the full study.

#### Conflict test

Immediately after experiencing each emotional treatment, the dogs’ preferences for local or global information were assessed once in a conflict test, approximately within 10–20 s of ending the treatment. This involved displaying two inconsistent test stimuli (Fig. 2b): one symbol with the same global element as the rewarded symbol, but with a local element never seen before (a preference for which would indicate a global preference) and another symbol showing the same local elements as the rewarded symbol, but with a global element never seen before (a preference for which would indicate a local preference). The dogs were always rewarded regardless of their choice. Each dog was tested twice in each emotional state, and right and left presentations of global and local choices were balanced across the repetitions.

#### Local and global discrimination test

To confirm that dogs could discriminate at local and global levels, and ensure that the emotional treatments did not impair the dog’s ability to discriminate at local and global levels, one local discrimination test and one global discrimination test^20^ were carried out directly after the conflict test, with an inter-trial interval of approximately 10–20 s. The procedure was identical to that of the conflict test but with the exception that the correct symbol differed from the rewarded symbol only at the global level for the global discrimination test (Fig. 2c) and only at the local level for the local discrimination test (Fig. 2d). Right and left presentations of correct local and global stimuli were randomized as well as the order of the local and global discrimination between the two repetitions. Only the choice of the correct local and global symbols was rewarded.

#### Motivation tests

A food motivation and a social motivation test were carried out once the local–global preference tests were completed to assess the general level of feeding and social motivation of each dog. This was done to account for individual differences in motivation toward food and social stimuli^45,46^ that could potentially influence how the dogs reacted in the emotional treatments in experiment 1 and 2, respectively. Motivation testing took place in a runway of 20 m long. For the feeding motivation test, a food bowl containing 1 piece of sausage, 1 piece of ham and 2 pieces of dry treats was placed at one end of the runway. For the social motivation test, a familiar person stood at the end of the runway and continued calling the dog’s name until the dog reached them. The time the dog took to run to the bowl or the person from the opposite end of the runway was recorded. The dog was allowed to eat the treats or was petted by the familiar person for 20 s. This process was repeated four times for each stimulus. The order of food and social motivation tests was randomized but balanced across the group.

### Heart rate and heart rate variability procedure

Dogs’ heart rates were recorded with Polar watches, model V800 Polar-Systems (© Polar Electro 2021), to obtain a non-invasive real-time measurement of HR and HRV. A belt with two electrodes was placed on the dog’s chest and connected to a watch-like data recorder. Electro gel was used to improve the skin-to-electrode contact. For each dog, a 15-min recording was taken (baseline) before the dogs received the emotional treatments and global–local testing. The baselines were recorded in the same room as where training and testing took place to ensure that the baselines were recorded in the same environment as the treatments.

Dogs were always very excited when coming into the training and testing room, so we could not record any baselines immediately before conducting the emotional treatments and tests. Therefore, baseline recordings were started about 5 min after the end of a training session to ensure a resting condition, during which the experimenter sat down quietly so that the dogs would relax and rest. The HR and HRV were then also recorded during each emotional treatment during the first repetition only because, unlike in the attentional scope test, these measures were not expected to be influenced by a potential side preference. HR and HRV were measured between 9:00 h and 11:00 h.

### Data analysis

Initial data exploration and statistical analysis were performed with the software R (version 4.0.2, 2020-06-22, copyright © 2020, the R Foundation for Statistical Computing) and R studio (version 1.3.1073, © 2009–2020 RStudio, PCB). Predicted means and confidence intervals (CI) were calculated using the package emmeans^47^ and plots and figures were generated using the package ggplot2^48^. All data are presented as predicted means ± (CI), unless stated otherwise.

#### Global–local preference data

The analysed variable was the choice made in the global–local preference test. Therefore, it was a binomial variable (0 = choice of global; 1 = choice of local) and the analysis was conducted on the proportion of local choices. A generalized linear mixed model (GLMM) with a logit link function was fitted using packages lme4^49^ and nmle^50^ with valence, arousal, repetition and their interactions, the side of presentation of the local choice (left or right), the assigned set of stimuli (set A or set B), and the sex of the dog as fixed effects, and the dog identification number as a random effect. To account for potential individual differences in the motivation for food or social stimuli, we included the average number of seconds to reach the end of the runway in the food and social motivation tests as covariates in the model, in experiment 1 and 2, respectively.

Due to the large number of potential fixed effects that could influence the proportion of local choices (e.g., valence, arousal, repetition, their interactions, sex, motivation, side and symbol of the stimuli), we used an information criterion approach for model selection. Akaike information criterion values (adjusted for small sample sizes, AICc) were calculated with the ‘dredge function’ with the Mumin package. The fixed effects in the top models (with a delta of 0) were included in the final statistical analysis. The top model in experiment 1 included a valence by repetition interaction and the main effect of sex (AICc = 98.1, delta = 0, weight = 0.312). The top model in experiment 2 included the main effects of valence and sex and motivation as a covariate (AICc = 110.3, delta = 0, weight = 0.038). Tukey corrected post-hoc test were conducted with the package emmeans. The data from the social test was over-dispersed, and therefore an observation level random effect was included to account for this^51^.

To compare the dogs’ abilities to discriminate on a local or global level, a generalized linear mixed model (GLMM) with a logit link function was conducted to compare the proportions correct (= 1) and incorrect (= 0) choices for the local and global discrimination test, fitting the discrimination test (local vs global) as a fixed effect and dog ID as a random effect.

#### Motivation test

The residuals from the motivation test were not normally distributed, so we assessed whether there was a difference in running speed between the social and food stimuli, and between the two sexes with a non-parametric Wilcoxon test.

#### Heart rate (HR) and heart rate variability (HRV)

The raw data were divided into two time periods for each treatment (one data point per dog and per period): a 15-min baseline period and a 45-s treatment period in repetition 1 (the last 45 s of the treatment exposure). The HRV analysis required the identification of outliers. The recordings were first checked by the algorithm AVEC (Algorithm-supported visual error correction) in R studio in order to highlight the outliers and these were afterwards manually removed in Excel 2016 (Windows 10). On average, 0.64% of the recordings were removed.

The cleaned heart rate recordings were then analysed with *ARTiiFACT* (Heart rate artefact processing and heart rate variability analysis software, version 2.13^52^) which associated a list of HRV indexes values to each time period of each treatment. *ARTiiFACT* software analysis yields all common time and frequency domain (using the FFT method) measures of vagally mediated HRV, including standard deviation of normal-to-normal R-R intervals (SDNN), Root Mean Square of Successive Differences (RMSSD), mean RR intervals, proportion of successive R–R intervals that differ by more than 50 ms (pNN50), High-Frequency (HF) absolute power, Low-Frequency absolute power (LF), and Very Low-Frequency absolute power (VLF). The VLF band was set to be 0.06 Hz, LF to be 0.24 Hz, and HF to be 1.04 Hz based on measurements done in dogs^53^.

These indexes were analysed in R with a GLMM, using packages lme4^49^ and lmerTest^54^, with emotional treatments and sex as fixed effects, the baseline values as a covariate and dog ID as a random effect. Data were log-transformed and assumptions of normality and homoscedasticity were assessed by graphical analysis of the residuals (LMERConvienienceFunction package^55^). Outliers with residuals greater than 2.5 units were excluded from the dataset (containing 119 data points). In experiment 1, we excluded 1 outlier from the SDNN, 1 outlier from the LF and 1 outlier from the VLF. In experiment 2, we excluded 1 outlier from the VLF. Tukey corrected post-hoc tests were conducted using the package emmeans^47^.

## Results

Only 10 out of 21 dogs passed all the training criteria. These 10 dogs were on average fully trained after 364 ± 27 (mean ± SE) trials, which is equivalent to 18 ± 1 (mean ± SE) sessions of 20 trials each.

In the motivation test, the average running speed in the runway was higher for the social reward (7.5 ± CI 3.9 s) than for the food reward (12.3 ± CI 4.4 s, Wilcoxon V = 460.5, P = 0.005).

### Experiment 1: Food reward stimuli

#### Global–local preference test

**Figure 4:**
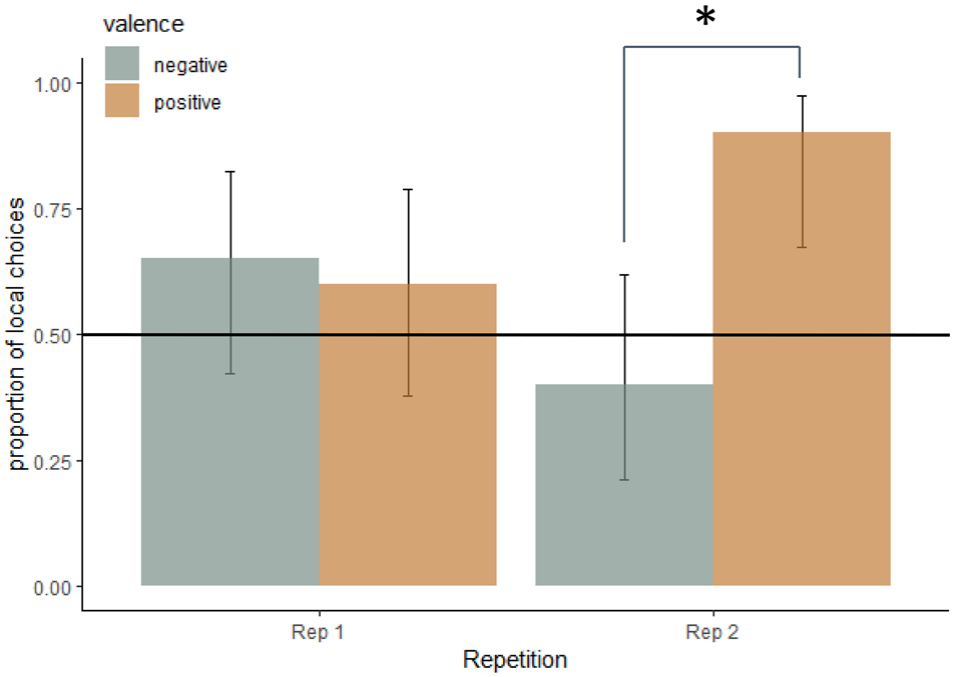
The effect of the valence and repetition (Table 1) on the proportion of local choices (predicted means ± CI) in the conflict test. Asterisk: indicates a difference between positive and negative valence conditions in the second repetition (P < 0.05). The line indicates 0.5 proportion of local choices.

There was a significant valence by repetition interaction on the proportion of local choices (F_(1,7.2)_ = 7.2, P = 0.03, Fig. 4), with the positive treatments leading to more local choices compared to the negative treatments in the second repetition (Tukey corrected posthoc test: P = 0.002). The data for each emotional treatment can be seen in Fig. 1 in the Supplementary Information.

There was no significant valence by arousal interaction. There was a tendency for an effect of sex on the proportion of local choices, with a higher proportion of female dogs choosing the local symbol (mean 0.78, lower CI = 0.62, upper CI = 0.87) compared to males (mean 0.5, lower CI = 0.35, upper CI = 0.64, F_(1,5)_ = 4.9, P = 0.08). None of the following variables; arousal, the test symbol set (A or B), side of presentation (left or right), or the animal’s running speed in the feeding motivation test affected the proportion of local choices.

We also determined whether the choice in each treatment was different from a 50–50 choice (indicating no preference for local or global elements) by calculating confidence intervals (CI). If the CI do not include 0.5, then we can conclude that there was a preference for local or global information. We found that the CI did not include 0.5 in the positive valence conditions in the second repetition (Fig. 4), suggesting that the positive conditions indeed induced a preference for the local elements. Also, the choices for the females did not include 0.5 in the confidence interval (see means given earlier in this section).

In the discrimination tests, to assess discrimination accuracy at the local and global levels, the dogs performed above chance at both the local (proportion of 0.78 correct choices, with a lower CI of 0.64 and an upper CI of 0.87) and the global level (proportion of 0.65 correct choices, with a lower CI of 0.50 and an upper CI of 0.77). Even though numerically dogs performed better in the local discrimination test, this difference was not statistically significant.

#### Heart rate and heart rate variability

**Figure 5:**
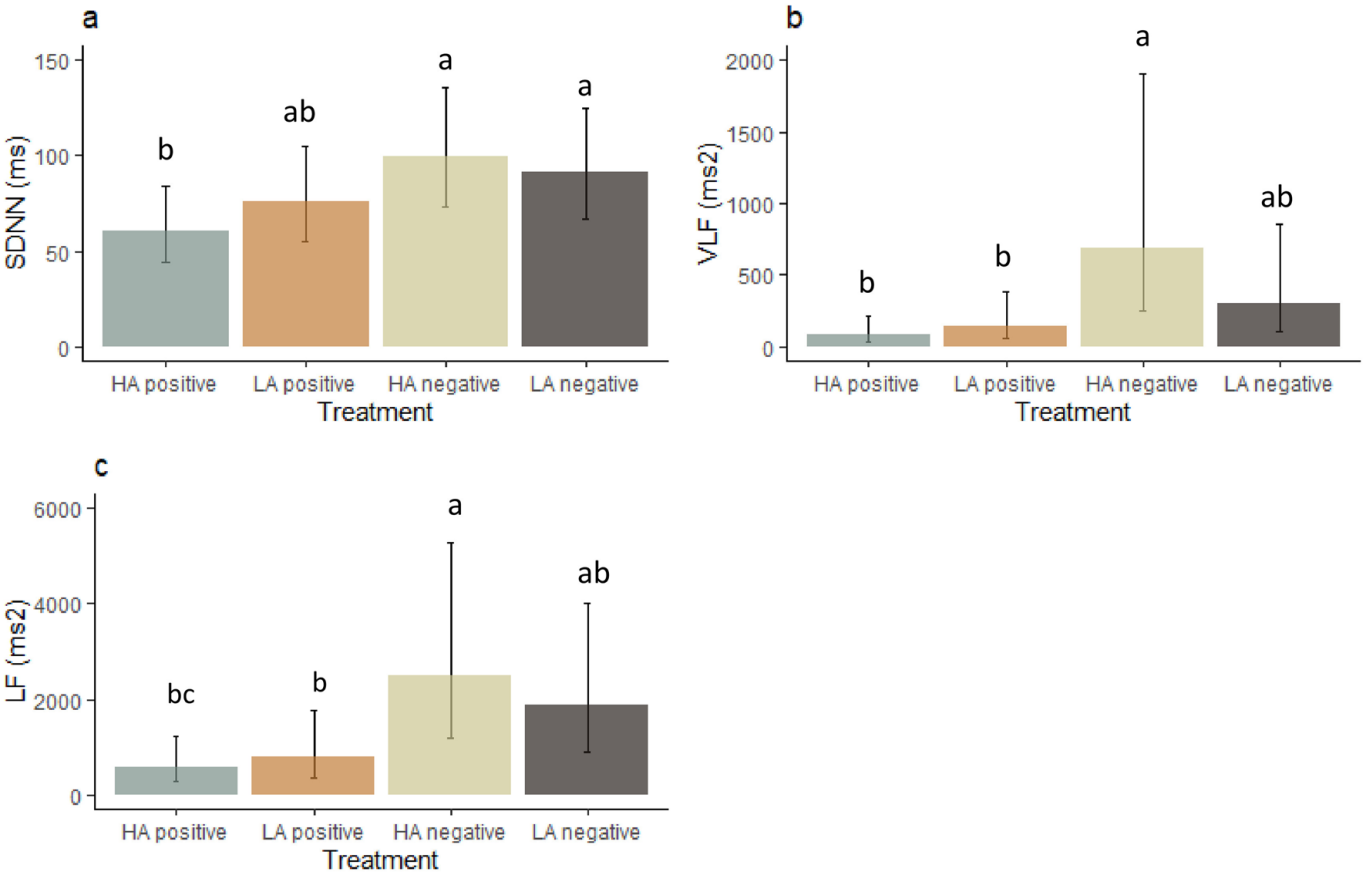
Effect of emotional treatment (Table 1) on heart rate variability indexes (mean ± CI), with (**a)** SDNN, (**b)** VLF and (**c)** LF. *HA* high arousal, *LA* low arousal. Bars that do not share the same letter are significantly different from each other (P < 0.05).

HRV was recorded on the first repetition only. The SDNN was affected by emotional treatment (F_(3, 25.3)_ = 4.8, P = 0.009, Fig. 5a). Post-hoc analysis showed that the SDNN was lower in the high arousal positive treatment than in the high arousal negative treatment (P = 0.009) and the low arousal negative treatment (P = 0.036).

The VLF was also influenced by emotional treatment (F_(3, 25.1)_ = 5.5, P = 0.005, Fig. 5b). The high arousal negative treatment had a higher VLF than the high arousal positive (P = 0.0041) and low arousal positive (P = 0.04) treatments. In addition, the VLF tended to be higher in female dogs (4476 ± 466 ms^2^) than in male dogs (2796 ± 305 ms^2^, F_(1, 6.7)_ = 4.1, P = 0.08).

The LF was affected by treatment (F_(3, 25.4)_ = 6.6, P = 0.002, Fig. 5c). Post-hoc analysis revealed that the LF was higher in the high arousal negative treatment than in the high arousal positive treatment (P = 0.004) and the low arousal positive treatment (P = 0.03). In addition, the low arousal negative treatment also had higher LF than the high arousal positive treatment (P = 0.02). No other HRV measure was affected by the treatments.

### Experiment 2: Social stimuli

#### Global–local preference test

**Figure 6:**
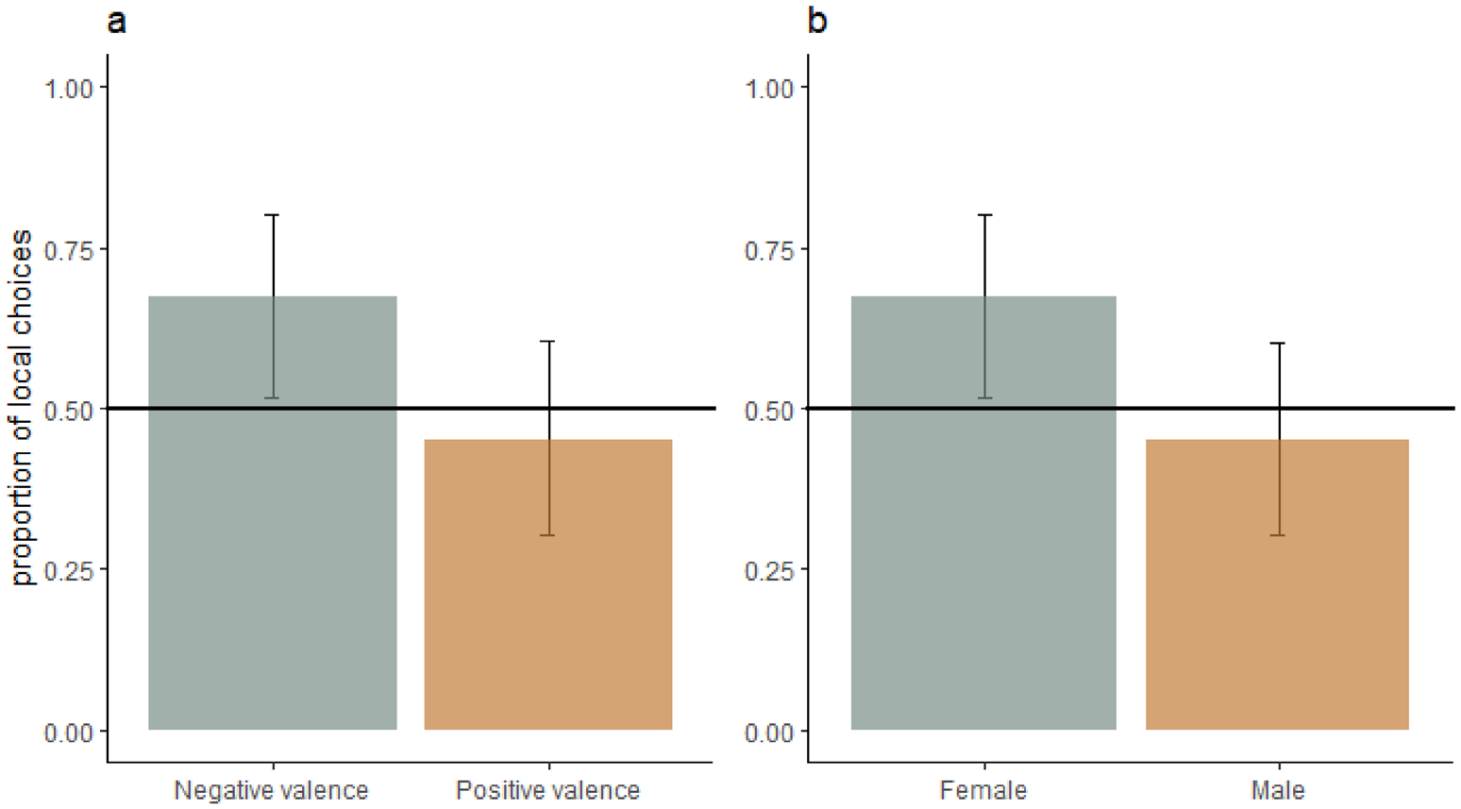
The effect of a. valence and b. sex on the proportion of local choices (predicted means ± CI) in the conflict test following the social emotional treatments (Table 2). The line indicates 0.5 proportion of local choices.

All dogs played with the experimenter in the high arousal positive treatment and wanted to be petted in the low arousal positive treatment. None of the factors fitted in the mixed model significantly affected the proportion of local choices (i.e., valence, arousal, repetition, sex, social motivation strength, set and side, Fig. 6). However, the CI did not include 0.5 proportion of local choice in the negative valence treatments, suggesting that these treatments induced a preference for local elements (Fig. 6). Also, the CI of the female dogs did not include 0.5 proportion of local choice, suggesting that the females more often made a local choice. The data for each emotional treatment can be seen in Fig. 2 in the supplementary information.

When assessing discrimination at the local and global levels only, the dogs performed significantly better in the local discrimination (proportion of 0.84 correct responses, with a lower CI of 0.74 and upper CI of 0.90 correct responses) than the global discrimination test (0.63 correct responses, with a lower CI of 0.51 and upper CI of 0.72 correct responses (F_(1, 8.8)_ = 8.8, P = 0.01).

#### Heart rate and heart rate variability

**Figure 7:**
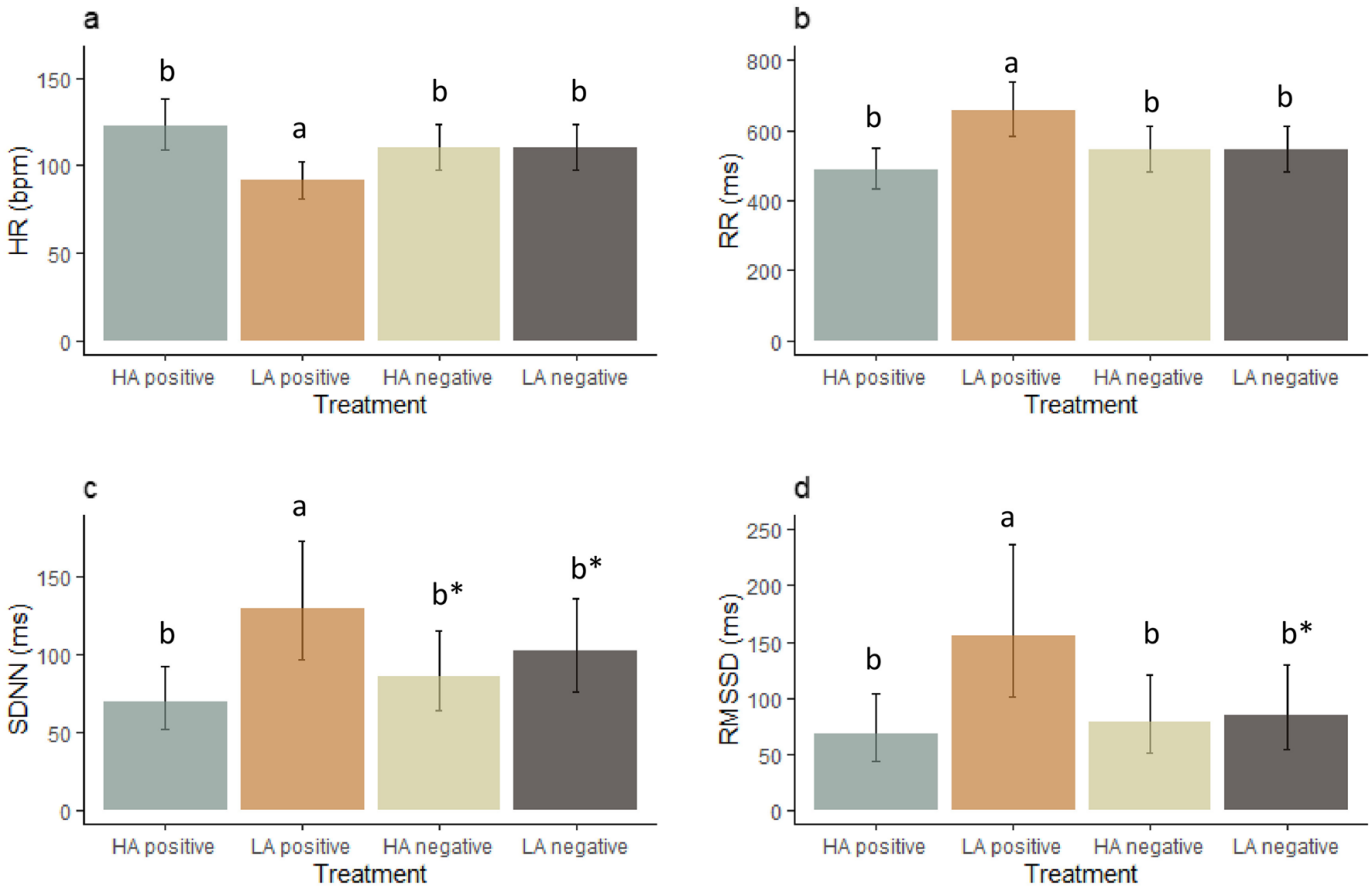
Effect of emotional treatment (Table 2) on heart rate variability indexes (mean ± CI), with (**a)** HR, (**b)** RR, (**c)** SDNN and (**d)** RMSSD. *HA* high arousal, *LA* low arousal. Bars that do not share the same letter are significantly different from each other (P < 0.05), Asterisk indicates a tendency for a difference (P < 0.1).

HR was influenced by treatment (F_(3, 27_ = 12.7, P < 0.001, Fig. 7a). Post-hoc analysis revealed that the dog’s HR responses were lower in the low arousal positive than in the high arousal positive (P < 0.001), high arousal negative (P = 0.037) and low arousal negative treatments (P = 0.0039).

The RR was also affected by treatment (F_(3, 27_ = 12.7, P < 0.001, Fig. 7b). The post-hoc tests showed that the dog’s RR response was higher in the low arousal positive treatment compared to the high arousal positive (P < 0.001), high arousal negative (P = 0.037) and low arousal negative treatments (P = 0.039).

The SDNN was also affected by treatment (F_(3, 27)_ = 6.5, P = 0.003, Fig. 7c). Dogs had a higher SDNN in the low arousal positive treatment than in the high arousal positive treatment (P = 0.002) and tended to be higher than the high arousal negative (P = 0.06) and low arousal negative treatments (P = 0.07).

The RMSSD was affected by treatment (F_(3, 27)_ = 5.0, P = 0.007, Fig. 7d). The RMSSD was higher in the low arousal positive treatment than in the high arousal positive (P = 0.007) and the high arousal negative treatments (P = 0.03), and tended to be higher than the low arousal negative treatment (P = 0.06).

**Figure 8:**
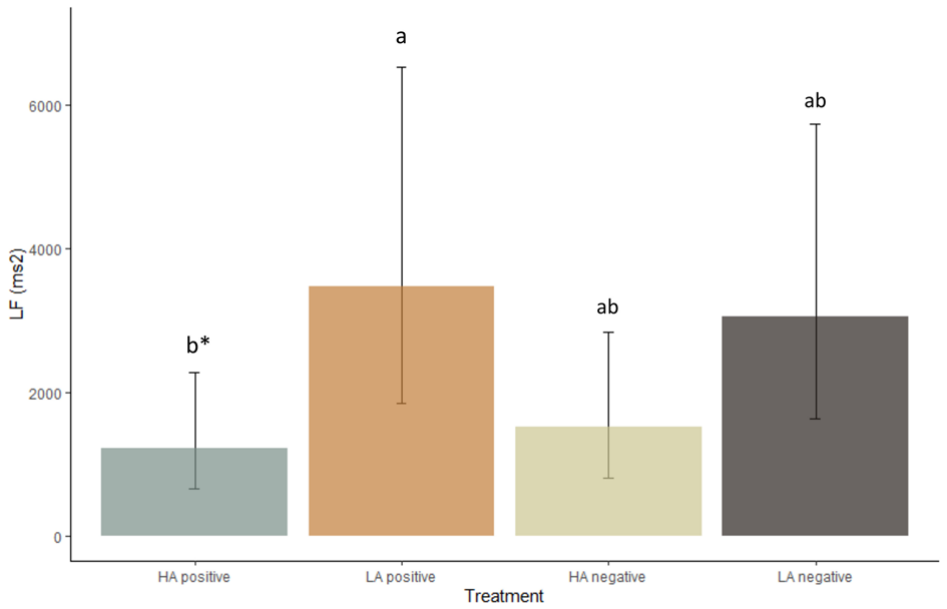
Effect of emotional treatment (Table 2) on heart rate variability indexes (mean ± CI), LF. *HA* high arousal, *LA* low arousal. Asterisk: bars that do not share the same letter tend to differ from each other (P < 0.1).

The LF was also influenced by treatment (F_(3, 27)_ = 3.1, P = 0.04, Fig. 8). The LF tended to be higher in the low arousal positive treatment compared to high arousal positive treatment (P = 0.07).

## Discussion

This study aimed to investigate attentional scope as a novel indicator of the emotional state in animals. In experiment 1, we found that dogs in the positive treatments, irrespective of arousal, showed a narrowing of attentional scope compared to dogs in the negative treatments, although only in the second repetition. In experiment 2, there was no significant effect of the treatments on attentional narrowing, although dogs appeared to express some degree of attentional narrowing in the negative compared to the positive treatments, irrespective of arousal. Although the results are not exactly as predicted, this is the first evidence that the emotional state can alter local and global preferences in animals, as has previously been shown in humans. It is possible that the induced arousal levels in some of the treatments were not as intended (e.g., were high instead of low) and that this potentially explains why the results were not always according to the predictions. HRV indexes confirmed that the positive treatments in experiment 1 induced the intended positive emotional states, although they may have reflected a difference in physical activity in experiment 2.

## Attentional scope

### Experiment 1. Food reward test

In the second repetition, dogs’ attention narrowed in the positively valenced conditions, irrespective of whether it was initial or prolonged access to food rewards. Therefore, it seems that the change in attentional scope mostly reflected the difference in emotional valence, regardless of arousal. A narrowing of attention when anticipating food treats (high arousal positive state) is supported by findings in humans^4,9,56^. However, we had expected attentional scope to broaden with prolonged access to food. One possible explanation for the lack of difference between the two positively valenced conditions could be that the dogs maintained a highly aroused anticipatory state even after receiving many food treats, and we may not have been successful in inducing a low arousal positive state. This is supported by the lack of difference in HR between the low and high arousal positive treatments. However, our sample size was relatively low due to the high dropout rate in the study, and we should therefore interpret our results with caution.

The high arousal negative treatment (frustrated approach) did not lead to the predicted narrowing of attention compared to the low arousal negative treatment, and dogs showed no clear preference for local or global information in the negative conditions. It is possible that the low arousal negative state did not have a sufficiently large impact on the emotional state of the dogs, and therefore we did not observe a clear local or global preference. In addition, we may not have been successful in lowering arousal in this condition, and dogs may still have been frustrated due to the absence of the reward^43,57^. Even though the HRV parameters were numerically lower in the low arousal condition compared to the high arousal condition, these differences did not reach statistical significance. Another potential explanation is that the test itself may have been rewarding for the dogs. Dogs where visibly excited when entering the testing room and were highly motivated to perform the training and tests. Therefore, the effect of any negative treatment may have disappeared as soon as the dogs performed the conflict test, which was always rewarded.

In contrast to the second repetition, the dogs had no clear preference for local or global elements in any emotional state in the first repetition. This temporal difference could be because the conflict stimuli had not been previously shown to the dogs, and the dogs may have been confused by the unfamiliar symbols, i.e. a response to novelty that masked initial preference^58^. This possibility is backed up by finding no such difference between repetitions in Experiment 2, when all the dogs had previously experienced the conflict stimuli. In future experiments, it may therefore be worthwhile to introduce dogs to the conflict stimuli as part of their training to reduce any potential influences of novelty.

### Experiment 2. Social interaction test

In experiment 2, there was no significant effect of the treatment on attentional narrowing. The proportion of local choices in the negative conditions was higher than 50% (i.e., the CI did not overlap with 0.5), suggesting a degree of attentional narrowing. Given the attentional narrowing following positive emotions that we observed in experiment 1, this seems rather contradictory. One possible explanation may be that we had unintentionally generated a high rather than a low arousal negative treatment. For example, it is possible that the no play treatment led to frustration—because dogs may have anticipated play, but were then ignored—leading to a degree of narrowing.

It is interesting that we did not observe a similar attentional narrowing in the positive treatments following the social stimuli. All dogs did engage in play with the experimenter, although we can perhaps argue that it may be more rewarding for dogs to play with an individual to whom they are socially attached (e.g. an owner or another dog)^59^, rather than a familiar experimenter. In the runway, dogs ran quicker toward the experimenter than toward the bowl of food. This suggests that the experimenter was perceived as rewarding, which is supported by other studies^33,60^. However, the faster running speed towards the experimenter is possibly because they kept calling the dog until the dog reached them, while the food bowl was placed down only once without any additional encouragement. The social motivation can therefore not directly be compared with the motivation for food in our study. In addition, the stimuli used to generate the high (play) and low (petting) positive treatments in the social test were not exactly the same (as in experiment 1), although they were related. This may also be a reason for the difference in results between experiment 1 and 2.

In both experiments, dogs performed better in the discrimination test at the local level than at the global level, although this did not reach statistical significance in experiment 1. Therefore, the dogs in our study seemed to have a general preference for local elements and a better ability to discriminate at the local level. A preference for local over global information has been found in a number of species^21–23^, although no clear preference was found previously in dogs^19^. The local preference in our study may be due to the small sample size, or may be related to either individual preferences^19^ or potentially the particular breed that we used. The beagle was originally bred for hunting. Selective breeding may have led to a high attentional focus on the details in the environment in order to facilitate the capturing of prey, which may explain the general preference for local stimuli. It is also possible that the spatial density of our elements was too low, which may also have increased the preference for local elements^22,61^. Therefore, in future studies it may be possible to improve discrimination at the global level by increasing the density of the symbols.

Female dogs tended to have a stronger preference for the local elements than males in both experiments. These results are supported by other studies, in which women have shown a better ability to process the local elements^62^. These sex differences may stem from hemispheric asymmetries, with the right hemisphere processing global elements, and left hemispheres processing local elements more efficiently^62,63^. However, no effect of the sex was found on the local/global preferences in previous studies in dogs^19,64^.

The global–local training task was challenging, and only 10 out of 21 dogs passed all learning criteria. Some dogs were too fearful, and would not approach the screen. Others did learn to approach the stimuli, but did not pass the learning criteria. We suspect that the age of the dog played a role, because no dog older than 8 years reached the criteria. It is well documented that cognitive abilities drastically reduce after 8 years of age^65^, and this may be why we could not train older dogs in this difficult task. Furthermore, the dogs used in this experiment were laboratory dogs kept in a kennel and were not familiar with training for visual discrimination, and the learning the task was challenging for them. However, a high drop-out rate during training was also reported in a previous study in pet dogs^19^. We recommend to use a much larger sample size in future studies to account for the high dropout rate.

### Heart rate and heart rate variability

In order to obtain complementary data that could confirm whether or not we had induced the intended emotional states, we also recorded HR and HRV parameters on the first repetition in each experiment.

The SDNN is a good predictor of overall HRV at the time of recording^28^. The SDNN was lower in the high arousal positive treatment compared to both negative treatments, suggesting that receiving treats was indeed perceived as positive by the dogs. The low arousal positive condition was somewhat intermediate. The LF indicates sympathetic activity and the VLF indicates sympatho-vagal balance^28,66^ and these measures are also correlated to the SDNN^67^. Both the LF and VLF were lower in the positive conditions, suggesting an activation of sympathetic neural activity and an inhibition of vagal activity^66,68^ associated with positive valence. This is in agreement with a previous study, in which the LF was lower when dogs anticipated a meatball compared to a less desired stimulus^30^. This supports that our positively valenced conditions were indeed experienced as positive by the dogs.

In experiment 2, the low arousal positive treatment reduced the HR compared to the other treatments, while it increased the RR, SDNN, RMSS and LF. The RMSSD represents vagal tonal regulatory activity^28^, and may reflect valance in dogs^30,33^. However, the increase in HRV indexes in our study is most likely due to the absence of physical activity involved in petting compared to the other treatments^69–71^. Nevertheless, this also suggests that the petting session may have been perceived as calming and thus lower in arousal compared to the other treatments^30,33,72^. Therefore, the HRV indexes seemed to reflect a change in physical activity in our study and, potentially, reduced arousal.

The time periods during which we recorded the HR and HRV were relatively short, and periods of at least 5 min are generally recommended, especially for frequency measures^28^. However, it is very difficult to keep a dog in a state of excitement or frustration longer than a minute or so because they will lose the excitement or give up trying to get the reward. Other studies have suggested that periods as short as 1 min are sufficient to measure HR, SDNN, and RMSSD as long as artefacts are carefully removed^73^, which we have done. Previous studies have used an even shorter period of time to record frequency measures of HRV in dogs (20 s)^30^. Furthermore, it has been shown that ultra-short term analysis of HRV is sufficient to assess mental stress^74^, with even frequency measures giving reliable results within 40–50 s, which is similar to our own study (45 s). However, we should be careful when interpreting the frequency measures due to the short time periods and low sample size in our study.

It was difficult to get an appropriate baseline for the HRV. We could not record a resting baseline in the home pen, because dogs were housed in groups and the other dogs may have affected the recordings. Neither could we record a baseline in a neutral emotional state immediately before testing, because dogs were always very excited when coming into the training and testing room. Therefore, we decided to record the baselines about 5 min after a training session when dogs were relaxed and resting, but in the same room as training and testing to control for the environment. In future experiment, it may be worthwhile to assess and compare different baseline HRVs (e.g., home pen, after training and before testing) with the treatment HRV.

We chose not to control the physical activity of the dogs during the treatments, in order to not influence the dogs’ responses in the local/global tests. However, while activity levels in the low and high arousal positive and in the high arousal negative conditions were roughly similar (i.e., pawing and walking around the hidden-food toy or pawing and walking around the rack covering it), physical activity was lower in the low arousal social treatment, and possibly also in the low arousal negative food treatment.

## Conclusion

This study provides the first indication that the emotional state may alter attentional scope in animals. Positive emotions originating from consuming food rewards led to a narrowing of attentional scope compared to negative emotions associated with the loss of a food reward. HRV indexes confirmed that obtaining food rewards was a positive experience compared to inaccessible food rewards, which was likely perceived as frustrating by the dogs. However, positive social interactions did not lead to attentional narrowing in a similar way as food rewards. Therefore, although the attentional scope methodology shows some promise in identifying differences in valence, further research with a larger sample size is necessary. Nevertheless, because this is a novel methodology to measure emotions in animals, this experiment can be used to guide better predictions in future studies.

## Supplementary Information

Supplementary Figures.

## Data Availability

All data is available here: https://osf.io/x2ue6/?view_only=0c8fad220dd34845baf07b047277cf61.
