# Supplementary material for: Investigating attentional scope as a novel indicator of emotional state in animals
Source: Sci Rep. 2022 Oct 19;12:17452. doi: 10.1038/s41598-022-21151-1 (PMC9582009; doi:10.1038/s41598-022-21151-1)
Supplement: Supplementary file 1 — Supplementary Figures. [file 41598_2022_21151_MOESM1_ESM.docx]

**Supplementary figures to the paper:**

**Investigating attentional scope as a novel indicator of emotional state in animals.**

*Anne Hamlaoui^1^, Linda Keeling^1^, Oliver Burman^2^, Else Verbeek^1*^*

^1^Swedish University of Agricultural Sciences, Department of Animal Environment and Health, Box 7068, 750 07, Uppsala, Sweden

^2^School of Life Sciences, University of Lincoln, Lincoln, UK.


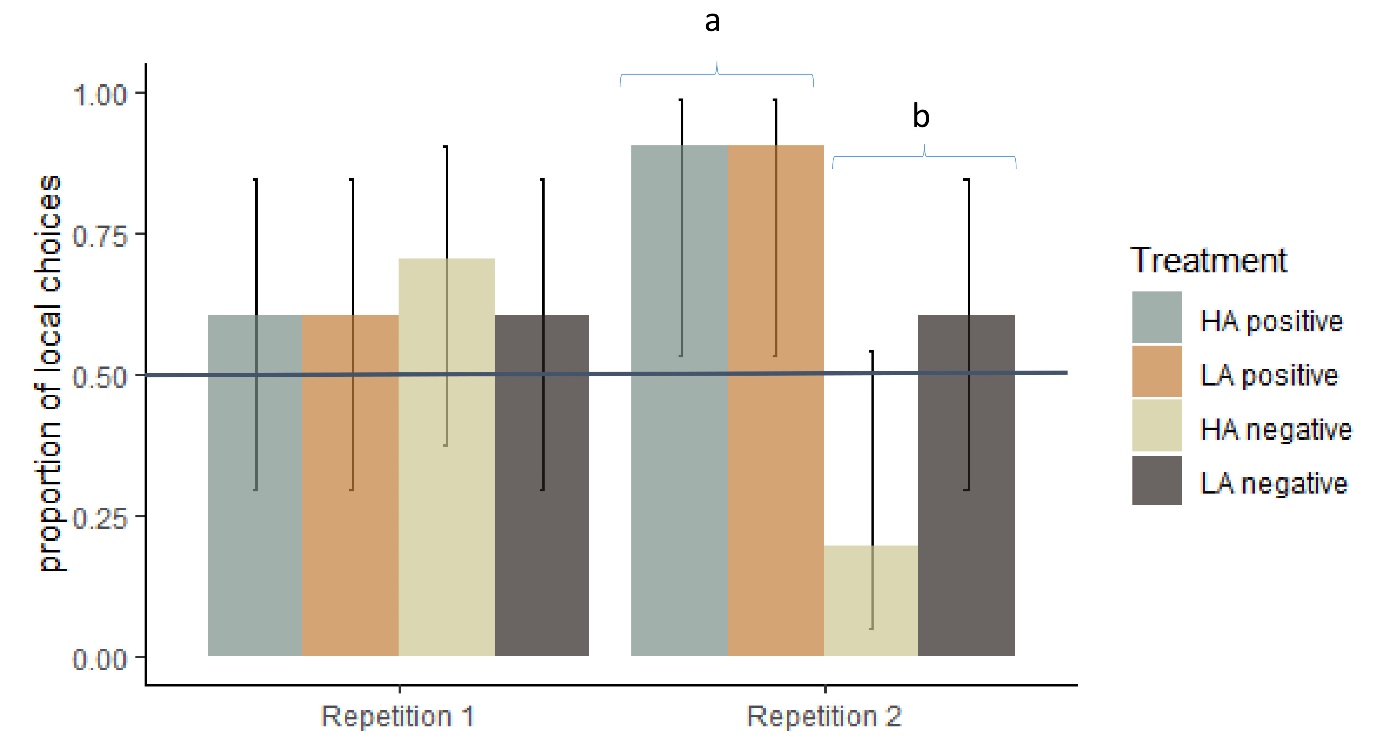
Figure 1. The effect of the emotional treatments (Table 1) on the proportion of local choices (predicted means ± CI) in the conflict test. HA stands for high arousal, LA stands for low arousal. Different letters indicate a difference between positive and negative valence conditions on the second repetition (P<0.05).


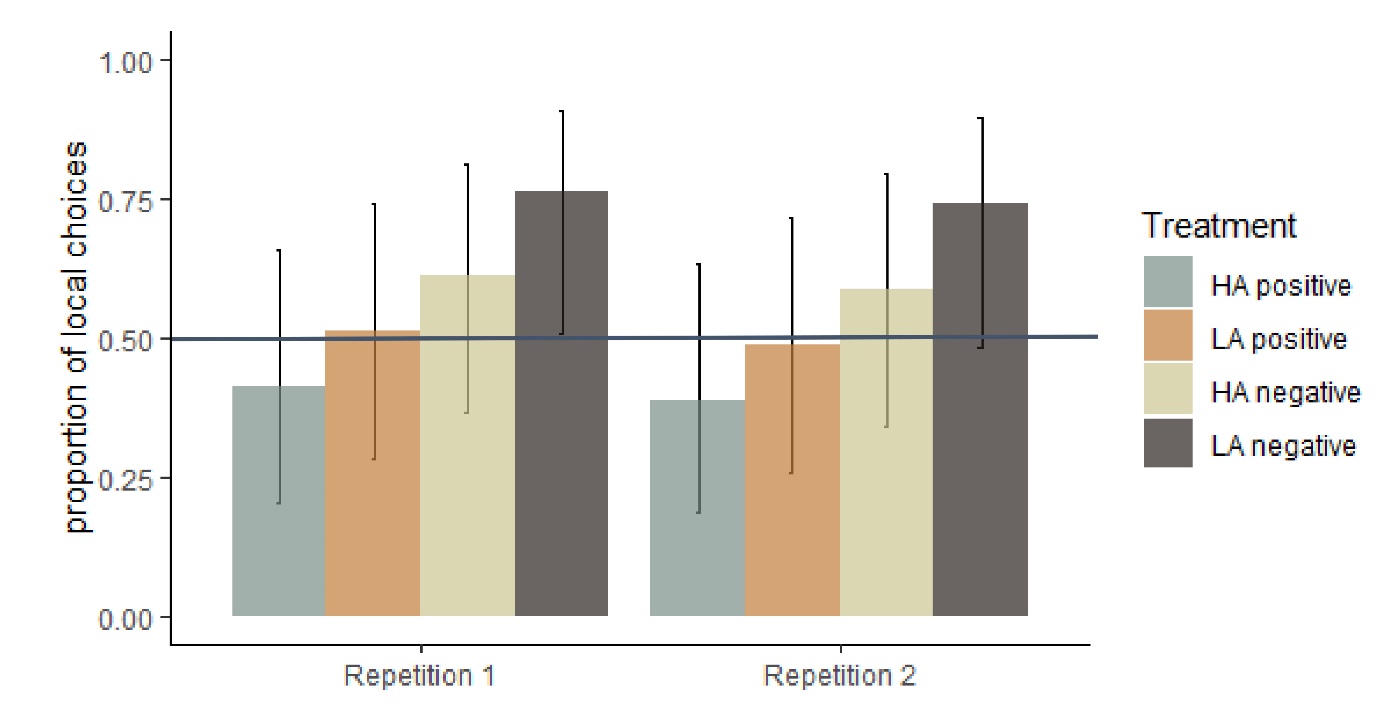


Figure 2. The effect of the emotional treatments (Table 2) on the proportion of local choices (predicted means ± CI) in the conflict test. HA stands for high arousal, LA stands for low arousal.
